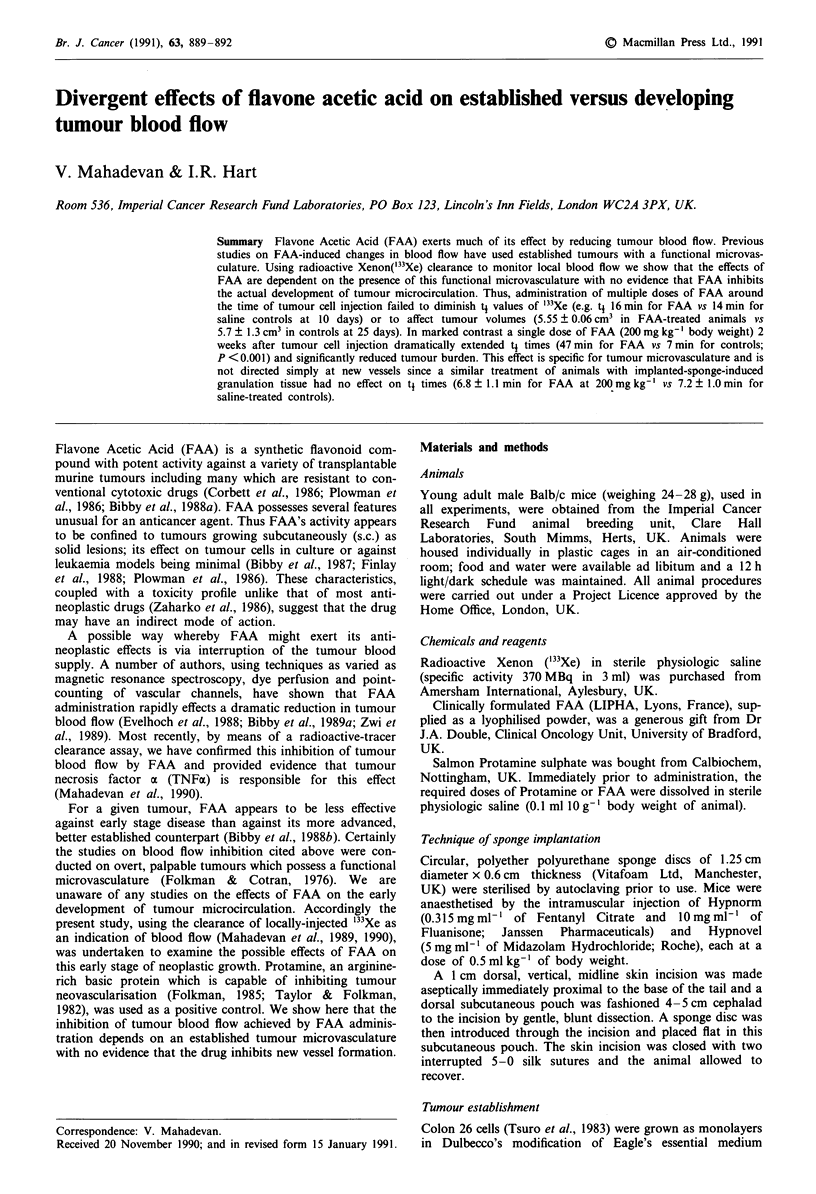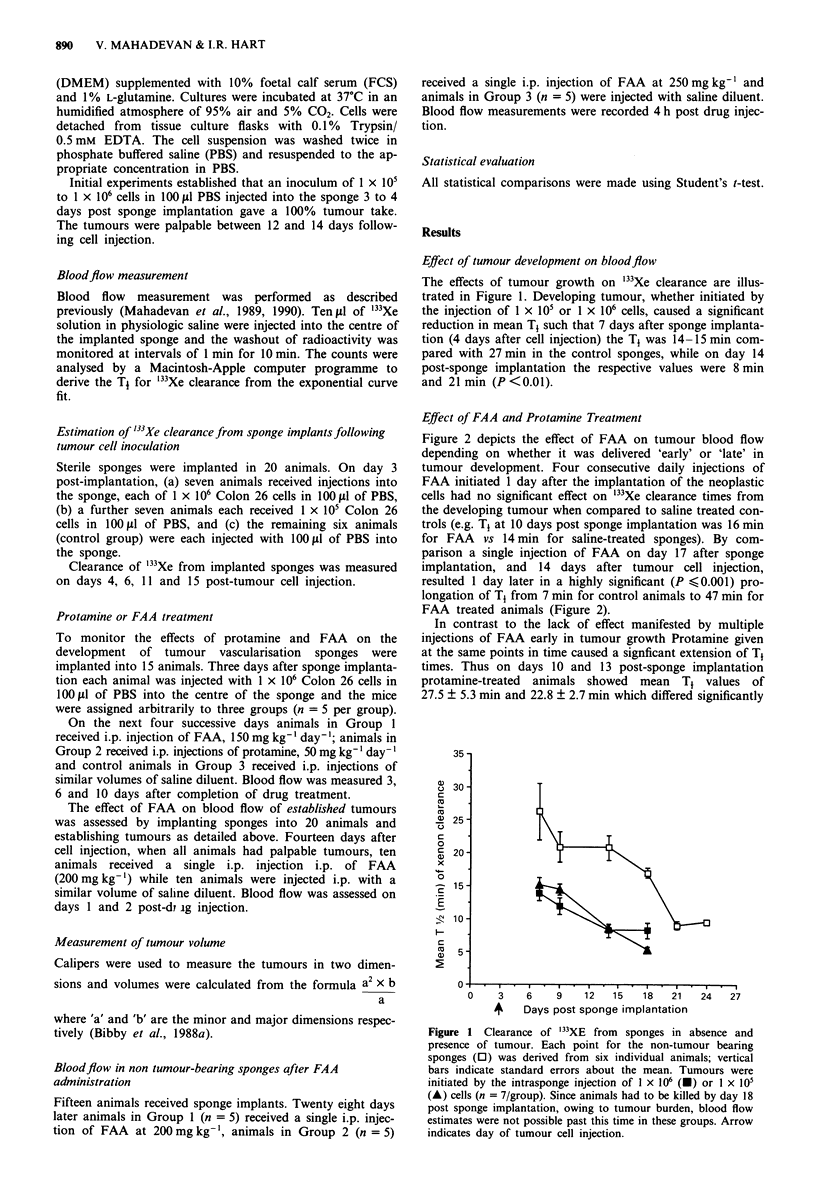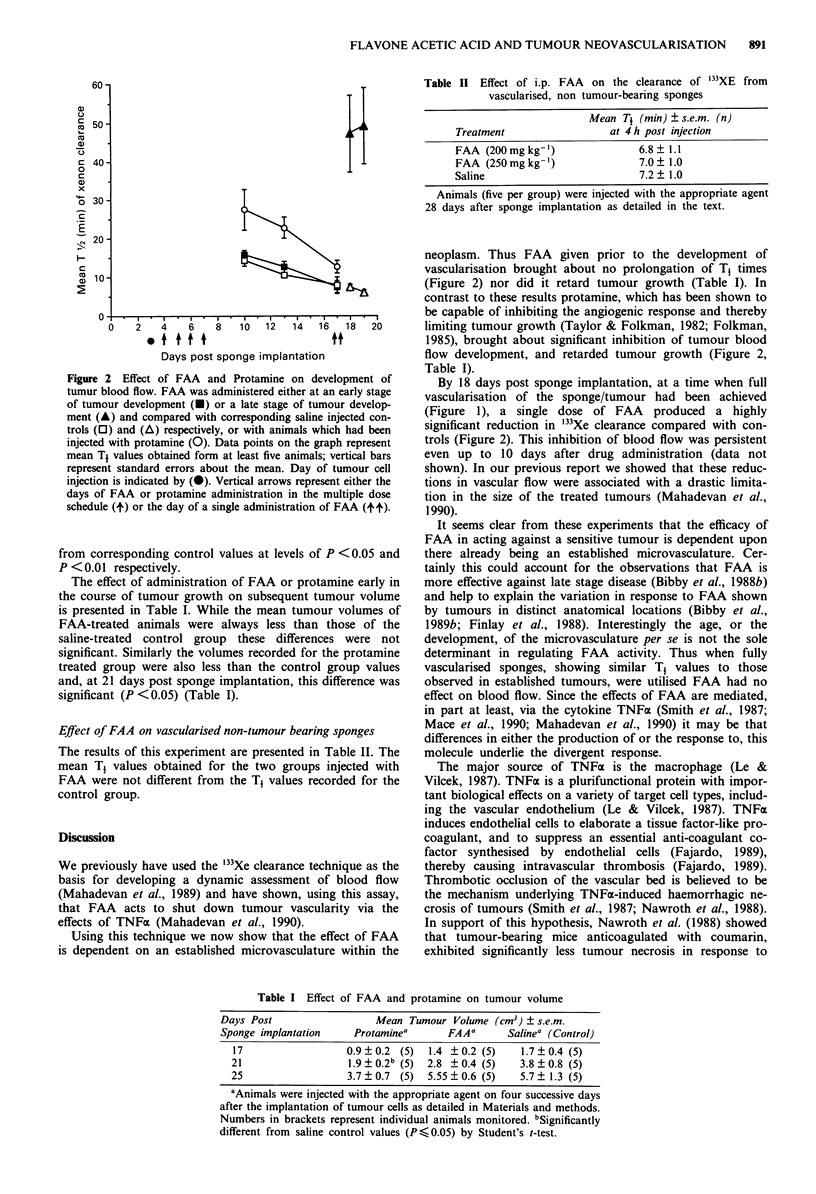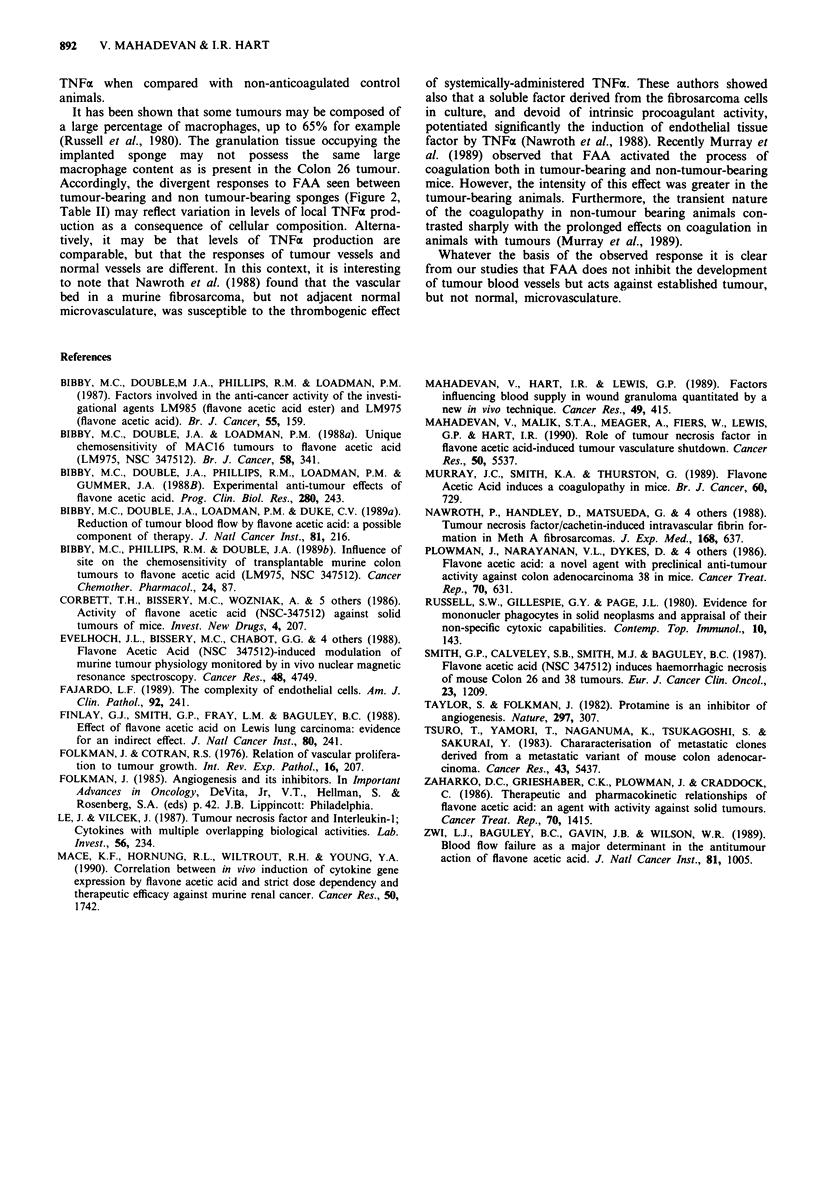# Divergent effects of flavone acetic acid on established versus developing tumour blood flow.

**DOI:** 10.1038/bjc.1991.195

**Published:** 1991-06

**Authors:** V. Mahadevan, I. R. Hart

**Affiliations:** Imperial Cancer Research Fund Laboratory, London, UK.

## Abstract

Flavone Acetic Acid (FAA) exerts much of its effect by reducing tumour blood flow. Previous studies on FAA-induced changes in blood flow have used established tumours with a functional microvasculature. Using radioactive Xenon(133Xe) clearance to monitor local blood flow we show that the effects of FAA are dependent on the presence of this functional microvasculature with no evidence that FAA inhibits the actual development of tumour microcirculation. Thus, administration of multiple doses of FAA around the time of tumour cell injection failed to diminish t1/2 values of 133Xe (e.g. t1/2 16 min for FAA vs 14 min for saline controls at 10 days) or to affect tumour volumes (5.55 +/- 0.06 cm3 in FAA-treated animals vs 5.7 +/- 1.3 cm3 in controls at 25 days). In marked contrast a single dose of FAA (200 mg kg-1 body weight) 2 weeks after tumour cell injection dramatically extended t1/2 times (47 min for FAA vs 7 min for controls; P less than 0.001) and significantly reduced tumour burden. This effect is specific for tumour microvasculature and is not directed simply at new vessels since a similar treatment of animals with implanted-sponge-induced granulation tissue had no effect on t1/2 times (6.8 +/- 1.1 min for FAA at 200 mg kg-1 vs 7.2 +/- 1.0 min for saline-treated controls.


Br. J. Cancer (1991), 63, 889-892 © Macmillan Press Ltd., 1991

### Divergent effects of flavone acetic acid on established versus developing tumour blood flow

V. Mahadevan & I.R. Hart

*Room 536, Imperial Cancer Research Fund Laboratories, PO Box 123, Lincoln's Inn Fields, London WC2A 3PX, UK.*

**Summary** Flavone Acetic Acid (FAA) exerts much of its effect by reducing tumour blood flow. Previous studies on FAA-induced changes in blood flow have used established tumours with a functional microvasculature. Using radioactive Xenon($^{133}$Xe) clearance to monitor local blood flow we show that the effects of FAA are dependent on the presence of this functional microvasculature with no evidence that FAA inhibits the actual development of tumour microcirculation. Thus, administration of multiple doses of FAA around the time of tumour cell injection failed to diminish $t_{\frac{1}{2}}$ values of $^{133}$Xe (e.g. $t_{\frac{1}{2}}$ 16 min for FAA *vs* 14 min for saline controls at 10 days) or to affect tumour volumes ($5.55 \pm 0.06\,cm^3$ in FAA-treated animals *vs* $5.7 \pm 1.3\,cm^3$ in controls at 25 days). In marked contrast a single dose of FAA (200 mg $kg^{-1}$ body weight) 2 weeks after tumour cell injection dramatically extended $t_{\frac{1}{2}}$ times (47 min for FAA *vs* 7 min for controls; $P < 0.001$) and significantly reduced tumour burden. This effect is specific for tumour microvasculature and is not directed simply at new vessels since a similar treatment of animals with implanted-sponge-induced granulation tissue had no effect on $t_{\frac{1}{2}}$ times ($6.8 \pm 1.1$ min for FAA at 200 mg $kg^{-1}$ *vs* $7.2 \pm 1.0$ min for saline-treated controls).

Flavone Acetic Acid (FAA) is a synthetic flavonoid compound with potent activity against a variety of transplantable murine tumours including many which are resistant to conventional cytotoxic drugs (Corbett *et al.*, 1986; Plowman *et al.*, 1986; Bibby *et al.*, 1988*a*). FAA possesses several features unusual for an anticancer agent. Thus FAA's activity appears to be confined to tumours growing subcutaneously (s.c.) as solid lesions; its effect on tumour cells in culture or against leukaemia models being minimal (Bibby *et al.*, 1987; Finlay *et al.*, 1988; Plowman *et al.*, 1986). These characteristics, coupled with a toxicity profile unlike that of most antineoplastic drugs (Zaharko *et al.*, 1986), suggest that the drug may have an indirect mode of action.

A possible way whereby FAA might exert its antineoplastic effects is via interruption of the tumour blood supply. A number of authors, using techniques as varied as magnetic resonance spectroscopy, dye perfusion and point-counting of vascular channels, have shown that FAA administration rapidly effects a dramatic reduction in tumour blood flow (Evelhoch *et al.*, 1988; Bibby *et al.*, 1989*a*; Zwi *et al.*, 1989). Most recently, by means of a radioactive-tracer clearance assay, we have confirmed this inhibition of tumour blood flow by FAA and provided evidence that tumour necrosis factor α (TNFα) is responsible for this effect (Mahadevan *et al.*, 1990).

For a given tumour, FAA appears to be less effective against early stage disease than against its more advanced, better established counterpart (Bibby *et al.*, 1988*b*). Certainly the studies on blood flow inhibition cited above were conducted on overt, palpable tumours which possess a functional microvasculature (Folkman & Cotran, 1976). We are unaware of any studies on the effects of FAA on the early development of tumour microcirculation. Accordingly the present study, using the clearance of locally-injected $^{133}$Xe as an indication of blood flow (Mahadevan *et al.*, 1989, 1990), was undertaken to examine the possible effects of FAA on this early stage of neoplastic growth. Protamine, an arginine-rich basic protein which is capable of inhibiting tumour neovascularisation (Folkman, 1985; Taylor & Folkman, 1982), was used as a positive control. We show here that the inhibition of tumour blood flow achieved by FAA administration depends on an established tumour microvasculature with no evidence that the drug inhibits new vessel formation.

Correspondence: V. Mahadevan.
Received 20 November 1990; and in revised form 15 January 1991.

### Materials and methods

### *Animals*

Young adult male Balb/c mice (weighing 24–28 g), used in all experiments, were obtained from the Imperial Cancer Research Fund animal breeding unit, Clare Hall Laboratories, South Mimms, Herts, UK. Animals were housed individually in plastic cages in an air-conditioned room; food and water were available ad libitum and a 12 h light/dark schedule was maintained. All animal procedures were carried out under a Project Licence approved by the Home Office, London, UK.

### *Chemicals and reagents*

Radioactive Xenon ($^{133}$Xe) in sterile physiologic saline (specific activity 370 MBq in 3 ml) was purchased from Amersham International, Aylesbury, UK.

Clinically formulated FAA (LIPHA, Lyons, France), supplied as a lyophilised powder, was a generous gift from Dr J.A. Double, Clinical Oncology Unit, University of Bradford, UK.

Salmon Protamine sulphate was bought from Calbiochem, Nottingham, UK. Immediately prior to administration, the required doses of Protamine or FAA were dissolved in sterile physiologic saline (0.1 ml 10 $g^{-1}$ body weight of animal).

### *Technique of sponge implantation*

Circular, polyether polyurethane sponge discs of 1.25 cm diameter × 0.6 cm thickness (Vitafoam Ltd, Manchester, UK) were sterilised by autoclaving prior to use. Mice were anaesthetised by the intramuscular injection of Hypnorm (0.315 mg $ml^{-1}$ of Fentanyl Citrate and 10 mg $ml^{-1}$ of Fluanisone; Janssen Pharmaceuticals) and Hypnovel (5 mg $ml^{-1}$ of Midazolam Hydrochloride; Roche), each at a dose of 0.5 ml $kg^{-1}$ of body weight.

A 1 cm dorsal, vertical, midline skin incision was made aseptically immediately proximal to the base of the tail and a dorsal subcutaneous pouch was fashioned 4–5 cm cephalad to the incision by gentle, blunt dissection. A sponge disc was then introduced through the incision and placed flat in this subcutaneous pouch. The skin incision was closed with two interrupted 5–0 silk sutures and the animal allowed to recover.

### *Tumour establishment*

Colon 26 cells (Tsuro *et al.*, 1983) were grown as monolayers in Dulbecco's modification of Eagle's essential medium

(DMEM) supplemented with 10% foetal calf serum (FCS) and 1% L-glutamine. Cultures were incubated at 37°C in an humidified atmosphere of 95% air and 5% $CO_2$. Cells were detached from tissue culture flasks with 0.1% Trypsin/ 0.5 mM EDTA. The cell suspension was washed twice in phosphate buffered saline (PBS) and resuspended to the appropriate concentration in PBS.

Initial experiments established that an inoculum of $1 \times 10^5$ to $1 \times 10^6$ cells in 100 µl PBS injected into the sponge 3 to 4 days post sponge implantation gave a 100% tumour take. The tumours were palpable between 12 and 14 days following cell injection.

*Blood flow measurement*

Blood flow measurement was performed as described previously (Mahadevan *et al.*, 1989, 1990). Ten µl of $^{133}$Xe solution in physiologic saline were injected into the centre of the implanted sponge and the washout of radioactivity was monitored at intervals of 1 min for 10 min. The counts were analysed by a Macintosh-Apple computer programme to derive the $T_{1/2}$ for $^{133}$Xe clearance from the exponential curve fit.

*Estimation of $^{133}$Xe clearance from sponge implants following tumour cell inoculation*

Sterile sponges were implanted in 20 animals. On day 3 post-implantation, (a) seven animals received injections into the sponge, each of $1 \times 10^6$ Colon 26 cells in 100 µl of PBS, (b) a further seven animals each received $1 \times 10^5$ Colon 26 cells in 100 µl of PBS, and (c) the remaining six animals (control group) were each injected with 100 µl of PBS into the sponge.

Clearance of $^{133}$Xe from implanted sponges was measured on days 4, 6, 11 and 15 post-tumour cell injection.

*Protamine or FAA treatment*

To monitor the effects of protamine and FAA on the development of tumour vascularisation sponges were implanted into 15 animals. Three days after sponge implantation each animal was injected with $1 \times 10^6$ Colon 26 cells in 100 µl of PBS into the centre of the sponge and the mice were assigned arbitrarily to three groups ($n = 5$ per group).

On the next four successive days animals in Group 1 received i.p. injection of FAA, 150 mg kg$^{-1}$ day$^{-1}$; animals in Group 2 received i.p. injections of protamine, 50 mg kg$^{-1}$ day$^{-1}$ and control animals in Group 3 received i.p. injections of similar volumes of saline diluent. Blood flow was measured 3, 6 and 10 days after completion of drug treatment.

The effect of FAA on blood flow of *established* tumours was assessed by implanting sponges into 20 animals and establishing tumours as detailed above. Fourteen days after cell injection, when all animals had palpable tumours, ten animals received a single i.p. injection i.p. of FAA (200 mg kg$^{-1}$) while ten animals were injected i.p. with a similar volume of saline diluent. Blood flow was assessed on days 1 and 2 post-drug injection.

*Measurement of tumour volume*

Calipers were used to measure the tumours in two dimensions and volumes were calculated from the formula $\frac{a^2 \times b}{a}$ where 'a' and 'b' are the minor and major dimensions respectively (Bibby *et al.*, 1988*a*).

*Blood flow in non tumour-bearing sponges after FAA administration*

Fifteen animals received sponge implants. Twenty eight days later animals in Group 1 ($n = 5$) received a single i.p. injection of FAA at 200 mg kg$^{-1}$, animals in Group 2 ($n = 5$) received a single i.p. injection of FAA at 250 mg kg$^{-1}$ and animals in Group 3 ($n = 5$) were injected with saline diluent. Blood flow measurements were recorded 4 h post drug injection.

*Statistical evaluation*

All statistical comparisons were made using Student's *t*-test.

### Results

*Effect of tumour development on blood flow*

The effects of tumour growth on $^{133}$Xe clearance are illustrated in Figure 1. Developing tumour, whether initiated by the injection of $1 \times 10^5$ or $1 \times 10^6$ cells, caused a significant reduction in mean $T_{1/2}$ such that 7 days after sponge implantation (4 days after cell injection) the $T_{1/2}$ was 14–15 min compared with 27 min in the control sponges, while on day 14 post-sponge implantation the respective values were 8 min and 21 min ($P < 0.01$).

*Effect of FAA and Protamine Treatment*

Figure 2 depicts the effect of FAA on tumour blood flow depending on whether it was delivered 'early' or 'late' in tumour development. Four consecutive daily injections of FAA initiated 1 day after the implantation of the neoplastic cells had no significant effect on $^{133}$Xe clearance times from the developing tumour when compared to saline treated controls (e.g. $T_{1/2}$ at 10 days post sponge implantation was 16 min for FAA *vs* 14 min for saline-treated sponges). By comparison a single injection of FAA on day 17 after sponge implantation, and 14 days after tumour cell injection, resulted 1 day later in a highly significant ($P \leqslant 0.001$) prolongation of $T_{1/2}$ from 7 min for control animals to 47 min for FAA treated animals (Figure 2).

In contrast to the lack of effect manifested by multiple injections of FAA early in tumour growth Protamine given at the same points in time caused a signficant extension of $T_{1/2}$ times. Thus on days 10 and 13 post-sponge implantation protamine-treated animals showed mean $T_{1/2}$ values of 27.5 ± 5.3 min and 22.8 ± 2.7 min which differed significantly

**Figure 1** Clearance of $^{133}$XE from sponges in absence and presence of tumour. Each point for the non-tumour bearing sponges (□) was derived from six individual animals; vertical bars indicate standard errors about the mean. Tumours were initiated by the intrasponge injection of $1 \times 10^6$ (■) or $1 \times 10^5$ (▲) cells ($n$ = 7/group). Since animals had to be killed by day 18 post sponge implantation, owing to tumour burden, blood flow estimates were not possible past this time in these groups. Arrow indicates day of tumour cell injection.

**Figure 2** Effect of FAA and Protamine on development of tumur blood flow. FAA was administered either at an early stage of tumour development (■) or a late stage of tumour development (▲) and compared with corresponding saline injected controls (□) and (△) respectively, or with animals which had been injected with protamine (○). Data points on the graph represent mean $T_{\frac{1}{2}}$ values obtained form at least five animals; vertical bars represent standard errors about the mean. Day of tumour cell injection is indicated by (●). Vertical arrows represent either the days of FAA or protamine administration in the multiple dose schedule (↑) or the day of a single administration of FAA (↑↑).

from corresponding control values at levels of $P<0.05$ and $P<0.01$ respectively.

The effect of administration of FAA or protamine early in the course of tumour growth on subsequent tumour volume is presented in Table I. While the mean tumour volumes of FAA-treated animals were always less than those of the saline-treated control group these differences were not significant. Similarly the volumes recorded for the protamine treated group were also less than the control group values and, at 21 days post sponge implantation, this difference was significant ($P<0.05$) (Table I).

*Effect of FAA on vascularised non-tumour bearing sponges*

The results of this experiment are presented in Table II. The mean $T_{\frac{1}{2}}$ values obtained for the two groups injected with FAA were not different from the $T_{\frac{1}{2}}$ values recorded for the control group.

### Discussion

We previously have used the $^{133}$Xe clearance technique as the basis for developing a dynamic assessment of blood flow (Mahadevan *et al.*, 1989) and have shown, using this assay, that FAA acts to shut down tumour vascularity via the effects of TNFα (Mahadevan *et al.*, 1990).

Using this technique we now show that the effect of FAA is dependent on an established microvasculature within the neoplasm. Thus FAA given prior to the development of vascularisation brought about no prolongation of $T_{\frac{1}{2}}$ times (Figure 2) nor did it retard tumour growth (Table I). In contrast to these results protamine, which has been shown to be capable of inhibiting the angiogenic response and thereby limiting tumour growth (Taylor & Folkman, 1982; Folkman, 1985), brought about significant inhibition of tumour blood flow development, and retarded tumour growth (Figure 2, Table I).

By 18 days post sponge implantation, at a time when full vascularisation of the sponge/tumour had been achieved (Figure 1), a single dose of FAA produced a highly significant reduction in $^{133}$Xe clearance compared with controls (Figure 2). This inhibition of blood flow was persistent even up to 10 days after drug administration (data not shown). In our previous report we showed that these reductions in vascular flow were associated with a drastic limitation in the size of the treated tumours (Mahadevan *et al.*, 1990).

It seems clear from these experiments that the efficacy of FAA in acting against a sensitive tumour is dependent upon there already being an established microvasculature. Certainly this could account for the observations that FAA is more effective against late stage disease (Bibby *et al.*, 1988*b*) and help to explain the variation in response to FAA shown by tumours in distinct anatomical locations (Bibby *et al.*, 1989*b*; Finlay *et al.*, 1988). Interestingly the age, or the development, of the microvasculature *per se* is not the sole determinant in regulating FAA activity. Thus when fully vascularised sponges, showing similar $T_{\frac{1}{2}}$ values to those observed in established tumours, were utilised FAA had no effect on blood flow. Since the effects of FAA are mediated, in part at least, via the cytokine TNFα (Smith *et al.*, 1987; Mace *et al.*, 1990; Mahadevan *et al.*, 1990) it may be that differences in either the production of or the response to, this molecule underlie the divergent response.

The major source of TNFα is the macrophage (Le & Vilcek, 1987). TNFα is a plurifunctional protein with important biological effects on a variety of target cell types, including the vascular endothelium (Le & Vilcek, 1987). TNFα induces endothelial cells to elaborate a tissue factor-like procoagulant, and to suppress an essential anti-coagulant cofactor synthesised by endothelial cells (Fajardo, 1989), thereby causing intravascular thrombosis (Fajardo, 1989). Thrombotic occlusion of the vascular bed is believed to be the mechanism underlying TNFα-induced haemorrhagic necrosis of tumours (Smith *et al.*, 1987; Nawroth *et al.*, 1988). In support of this hypothesis, Nawroth *et al.* (1988) showed that tumour-bearing mice anticoagulated with coumarin, exhibited significantly less tumour necrosis in response to

**Table II** Effect of i.p. FAA on the clearance of $^{133}$XE from vascularised, non tumour-bearing sponges

| *Treatment* | *Mean $T_{\frac{1}{2}}$ (min) ± s.e.m. (n) at 4 h post injection* |
|---|---|
| FAA (200 mg $kg^{-1}$) | 6.8 ± 1.1 |
| FAA (250 mg $kg^{-1}$) | 7.0 ± 1.0 |
| Saline | 7.2 ± 1.0 |

Animals (five per group) were injected with the appropriate agent 28 days after sponge implantation as detailed in the text.

**Table I** Effect of FAA and protamine on tumour volume

| *Days Post Sponge implantation* | *Mean Tumour Volume ($cm^3$) ± s.e.m.* | | |
|---|---|---|---|
| | *Protamine[a]* | *FAA[a]* | *Saline[a] (Control)* |
| 17 | 0.9 ± 0.2 (5) | 1.4 ± 0.2 (5) | 1.7 ± 0.4 (5) |
| 21 | 1.9 ± 0.2[b] (5) | 2.8 ± 0.4 (5) | 3.8 ± 0.8 (5) |
| 25 | 3.7 ± 0.7 (5) | 5.55 ± 0.6 (5) | 5.7 ± 1.3 (5) |

[a]Animals were injected with the appropriate agent on four successive days after the implantation of tumour cells as detailed in Materials and methods. Numbers in brackets represent individual animals monitored. [b]Significantly different from saline control values ($P \leqslant 0.05$) by Student's *t*-test.

TNFα when compared with non-anticoagulated control animals.

It has been shown that some tumours may be composed of a large percentage of macrophages, up to 65% for example (Russell *et al.*, 1980). The granulation tissue occupying the implanted sponge may not possess the same large macrophage content as is present in the Colon 26 tumour. Accordingly, the divergent responses to FAA seen between tumour-bearing and non tumour-bearing sponges (Figure 2, Table II) may reflect variation in levels of local TNFα production as a consequence of cellular composition. Alternatively, it may be that levels of TNFα production are comparable, but that the responses of tumour vessels and normal vessels are different. In this context, it is interesting to note that Nawroth *et al.* (1988) found that the vascular bed in a murine fibrosarcoma, but not adjacent normal microvasculature, was susceptible to the thrombogenic effect of systemically-administered TNFα. These authors showed also that a soluble factor derived from the fibrosarcoma cells in culture, and devoid of intrinsic procoagulant activity, potentiated significantly the induction of endothelial tissue factor by TNFα (Nawroth *et al.*, 1988). Recently Murray *et al.* (1989) observed that FAA activated the process of coagulation both in tumour-bearing and non-tumour-bearing mice. However, the intensity of this effect was greater in the tumour-bearing animals. Furthermore, the transient nature of the coagulopathy in non-tumour bearing animals contrasted sharply with the prolonged effects on coagulation in animals with tumours (Murray *et al.*, 1989).

Whatever the basis of the observed response it is clear from our studies that FAA does not inhibit the development of tumour blood vessels but acts against established tumour, but not normal, microvasculature.

### References

BIBBY, M.C., DOUBLE,M J.A., PHILLIPS, R.M. & LOADMAN, P.M. (1987). Factors involved in the anti-cancer activity of the investigational agents LM985 (flavone acetic acid ester) and LM975 (flavone acetic acid). *Br. J. Cancer*, **55**, 159.

BIBBY, M.C., DOUBLE, J.A. & LOADMAN, P.M. (1988*a*). Unique chemosensitivity of MAC16 tumours to flavone acetic acid (LM975, NSC 347512). *Br. J. Cancer*, **58**, 341.

BIBBY, M.C., DOUBLE, J.A., PHILLIPS, R.M., LOADMAN, P.M. & GUMMER, J.A. (1988*B*). Experimental anti-tumour effects of flavone acetic acid. *Prog. Clin. Biol. Res.*, **280**, 243.

BIBBY, M.C., DOUBLE, J.A., LOADMAN, P.M. & DUKE, C.V. (1989*a*). Reduction of tumour blood flow by flavone acetic acid: a possible component of therapy. *J. Natl Cancer Inst.*, **81**, 216.

BIBBY, M.C., PHILLIPS, R.M. & DOUBLE, J.A. (1989*b*). Influence of site on the chemosensitivity of transplantable murine colon tumours to flavone acetic acid (LM975, NSC 347512). *Cancer Chemother. Pharmacol.*, **24**, 87.

CORBETT, T.H., BISSERY, M.C., WOZNIAK, A. & 5 others (1986). Activity of flavone acetic acid (NSC-347512) against solid tumours of mice. *Invest. New Drugs*, **4**, 207.

EVELHOCH, J.L., BISSERY, M.C., CHABOT, G.G. & 4 others (1988). Flavone Acetic Acid (NSC 347512)-induced modulation of murine tumour physiology monitored by in vivo nuclear magnetic resonance spectroscopy. *Cancer Res.*, **48**, 4749.

FAJARDO, L.F. (1989). The complexity of endothelial cells. *Am. J. Clin. Pathol.*, **92**, 241.

FINLAY, G.J., SMITH, G.P., FRAY, L.M. & BAGULEY, B.C. (1988). Effect of flavone acetic acid on Lewis lung carcinoma: evidence for an indirect effect. *J. Natl Cancer Inst.*, **80**, 241.

FOLKMAN, J. & COTRAN, R.S. (1976). Relation of vascular proliferation to tumour growth. *Int. Rev. Exp. Pathol.*, **16**, 207.

FOLKMAN, J. (1985). Angiogenesis and its inhibitors. In *Important Advances in Oncology*, DeVita, Jr, V.T., Hellman, S. & Rosenberg, S.A. (eds) p. 42. J.B. Lippincott: Philadelphia.

LE, J. & VILCEK, J. (1987). Tumour necrosis factor and Interleukin-1; Cytokines with multiple overlapping biological activities. *Lab. Invest.*, **56**, 234.

MACE, K.F., HORNUNG, R.L., WILTROUT, R.H. & YOUNG, Y.A. (1990). Correlation between *in vivo* induction of cytokine gene expression by flavone acetic acid and strict dose dependency and therapeutic efficacy against murine renal cancer. *Cancer Res.*, **50**, 1742.

MAHADEVAN, V., HART, I.R. & LEWIS, G.P. (1989). Factors influencing blood supply in wound granuloma quantitated by a new *in vivo* technique. *Cancer Res.*, **49**, 415.

MAHADEVAN, V., MALIK, S.T.A., MEAGER, A., FIERS, W., LEWIS, G.P. & HART, I.R. (1990). Role of tumour necrosis factor in flavone acetic acid-induced tumour vasculature shutdown. *Cancer Res.*, **50**, 5537.

MURRAY, J.C., SMITH, K.A. & THURSTON, G. (1989). Flavone Acetic Acid induces a coagulopathy in mice. *Br. J. Cancer*, **60**, 729.

NAWROTH, P., HANDLEY, D., MATSUEDA, G. & 4 others (1988). Tumour necrosis factor/cachetin-induced intravascular fibrin formation in Meth A fibrosarcomas. *J. Exp. Med.*, **168**, 637.

PLOWMAN, J., NARAYANAN, V.L., DYKES, D. & 4 others (1986). Flavone acetic acid: a novel agent with preclinical anti-tumour activity against colon adenocarcinoma 38 in mice. *Cancer Treat. Rep.*, **70**, 631.

RUSSELL, S.W., GILLESPIE, G.Y. & PAGE, J.L. (1980). Evidence for mononucler phagocytes in solid neoplasms and appraisal of their non-specific cytoxic capabilities. *Contemp. Top. Immunol.*, **10**, 143.

SMITH, G.P., CALVELEY, S.B., SMITH, M.J. & BAGULEY, B.C. (1987). Flavone acetic acid (NSC 347512) induces haemorrhagic necrosis of mouse Colon 26 and 38 tumours. *Eur. J. Cancer Clin. Oncol.*, **23**, 1209.

TAYLOR, S. & FOLKMAN, J. (1982). Protamine is an inhibitor of angiogenesis. *Nature*, **297**, 307.

TSURO, T., YAMORI, T., NAGANUMA, K., TSUKAGOSHI, S. & SAKURAI, Y. (1983). Chararacterisation of metastatic clones derived from a metastatic variant of mouse colon adenocarcinoma. *Cancer Res.*, **43**, 5437.

ZAHARKO, D.C., GRIESHABER, C.K., PLOWMAN, J. & CRADDOCK, C. (1986). Therapeutic and pharmacokinetic relationships of flavone acetic acid: an agent with activity against solid tumours. *Cancer Treat. Rep.*, **70**, 1415.

ZWI, L.J., BAGULEY, B.C., GAVIN, J.B. & WILSON, W.R. (1989). Blood flow failure as a major determinant in the antitumour action of flavone acetic acid. *J. Natl Cancer Inst.*, **81**, 1005.